# Age and comorbidities do not affect short-term outcomes after laparoscopic rectal cancer resection in elderly patients. A multi-institutional cohort study in 287 patients

**DOI:** 10.1007/s13304-021-00990-z

**Published:** 2021-02-14

**Authors:** Roberto Peltrini, Nicola Imperatore, Filippo Carannante, Diego Cuccurullo, Gabriella Teresa Capolupo, Umberto Bracale, Marco Caricato, Francesco Corcione

**Affiliations:** 1 Department of Public Health, School of Medicine and Surgery, University of Naples Federico II, Via Pansini 5, 80131 Naples, Italy; 2 Department of Clinical Medicine and Surgery, University of Naples Federico II, Naples, Italy; 3 Gastroenterology and Endoscopy Unit, AORN Antonio Cardarelli, Naples, Italy; 4 Colorectal Surgery Unit, Campus BioMedico University Hospital, Rome, Italy; 5 General Surgery Unit, Monaldi Hospital, Naples, Italy

**Keywords:** Rectal cancer, Laparoscopy, Elderly, Surgery, Postoperative complications, Short-term outcomes

## Abstract

Postoperative complications and mortality rates after rectal cancer surgery are higher in elderly than in non-elderly patients. The aim of this study is to evaluate whether, like in open surgery, age and comorbidities affect postoperative outcomes limiting the benefits of a laparoscopic approach. Between April 2011 and July 2020, data of 287 patients with rectal cancer submitted to laparoscopic rectal resection from different institutions were collected in an electronic database and were categorized into two groups: < 75 years and ≥ 75 years of age. Perioperative data and short-term outcomes were compared between these groups. Risk factors for postoperative complications were determined on multivariate analysis, including age groups and previous comorbidities as variables. Seventy-seven elderly patients had both higher ASA scores (*p* < 0.001) and cardiovascular disease rates (*p* = 0.02) compared with 210 non-elderly patients. There were no significative differences between groups in terms of overall postoperative complications (*p* = 0.3), number of patients with complications (*p* = 0.2), length of stay (*p* = 0.2) and death during hospitalization (*p* = 0.9). The only independent variables correlated with postoperative morbidity were male gender (OR 2.56; 95% CI 1.53–3.68, *p* < 0.01) and low-medium localization of the tumor (OR 2.12; 75% CI 1.43–4.21, *p* < 0.01). Although older people are more frail patients, short-term postoperative outcomes in patients ≥ 75 years of age were similar to those of younger patients after laparoscopic surgery for rectal cancer. Elderly patients benefit from laparoscopic rectal resection as well as non-elderly patient, despite advanced age and comorbidities.

## Introduction

Treatment of locally advanced mid or low rectal cancer is based on neoadjuvant chemoradiation followed by total mesorectal excision (TME) [1]. However, there is no consensus about the optimum surgical management of older patients [2, 3]. Elderly people are an heterogeneous subset of patients. Indeed, while it is considered appropriate to apply the same 'standard of care' to this category of patients, the increased risk of postoperative complications and mortality must be considered in patients with coexisting comorbidities and reduced physiological reserve capacity [4]. In this regard, advanced age should not represent itself a reason for exclusion of patients from radical surgery, but rather the frailty of these patients themselves is to be considered a primary risk factor [2, 3].

Several randomized controlled trials (RCTs) and meta-analyses [5–9] demonstrated the safety of laparoscopic rectal cancer surgery, better functional recovery and oncological outcomes comparable to open surgery. Despite it is well known that the number of elderly patients is poorly represented in clinical trials, underestimating the 'real-life data' [10–12], laparoscopic colorectal surgery has significant advantages in short-term outcomes also in the elderly population [13–15].

However, advanced age and comorbidities increase mortality and occurrence of complications after surgery for rectal cancer [3]. In fact, the preoperative comorbidity rate, which makes the patient vulnerable to postoperative complications, is highest after age 75 [16, 17] and this value may increase in the future because of demographic increase of an aging population and the increase in life expectancy [3].

For this reason, several authors investigated whether a laparoscopic approach in colorectal cancer surgery is as safe and feasible in elderly as in relatively younger patients [18] showing a significative higher overall complication rate in the old people, just like in open surgery [3]. However, focus on laparoscopic rectal resection is limited [19, 20] and these studies are mostly single center without discriminating outcomes between colon and rectal surgery [21–30], despite rectal surgery is associated with higher complication rate as well as in open surgery [21, 31–33]. Furthermore, most of the studies concern Eastern populations [19, 21, 23–28], characterized by lower body mass index (BMI) values and a lower preoperative comorbidity rate than Western population, as well as a limited use of neoadjuvant chemoradiation in locally advanced low rectal cancer, according to Japanese Society for Cancer of the Colon and Rectum Guidelines for the treatment of colorectal cancer [34]. Finally, the chronological cut-off is often 70 years, although it is now widely accepted that for the definition of elderly patient it should be of 75 years [35].

Because of the paucity of data concerning rectal cancer treatment and heterogeneous studies on the issue, the aim of this study is to assess the safety of laparoscopic approach for the treatment of rectal cancer in elderly patients and the impact of age on postoperative clinical outcomes, by comparing the characteristics and results of a retrospective analysis with those of a relatively younger patient group. Additionally, the study aim to evaluate age and comorbidities as potential independent risk factor for postoperative complications.

## Materials and methods

### Study design

This is a retrospective multi-institutional study. Between April 2011 and July 2020, patients scheduled for rectal cancer surgery were evaluated in three centers (Federico II University Hospital—Minimally Invasive General and Oncological Surgery Unit, Monaldi Hospital in Naples, along with Colorectal Surgery Unit of Campus Biomedico University in Rome) all considered centers with high specific case volume and with consolidated experience in the minimally invasive approach. Data from prospectively maintained electronic databases were retrieved into a comprehensive dataset.

All patients submitted to surgery with laparoscopic approach were divided into two cohorts: elderly patients (age ≥ 75 years) and non-elderly patients (age < 75 years). This cut-off was used in this study since age ≥ 75 years is considered a significant risk factor for postoperative complications in colorectal surgery [25, 36, 37], being also in accordance with a recent redefinition of age limits for elderly patients [38].

The primary endpoint of the study was overall rate of postoperative complications in the two groups and to investigate whether age is in itself a risk factor related to postoperative morbidity after laparoscopic anterior resection of rectal cancer. Secondary endpoints were the detection of any other difference between the two groups regarding short-term postoperative outcomes and the identification of predictors of complications.

This study was conducted according to the STROBE Guidelines [39].

### Patient selection and data collection

**Fig. 1:**
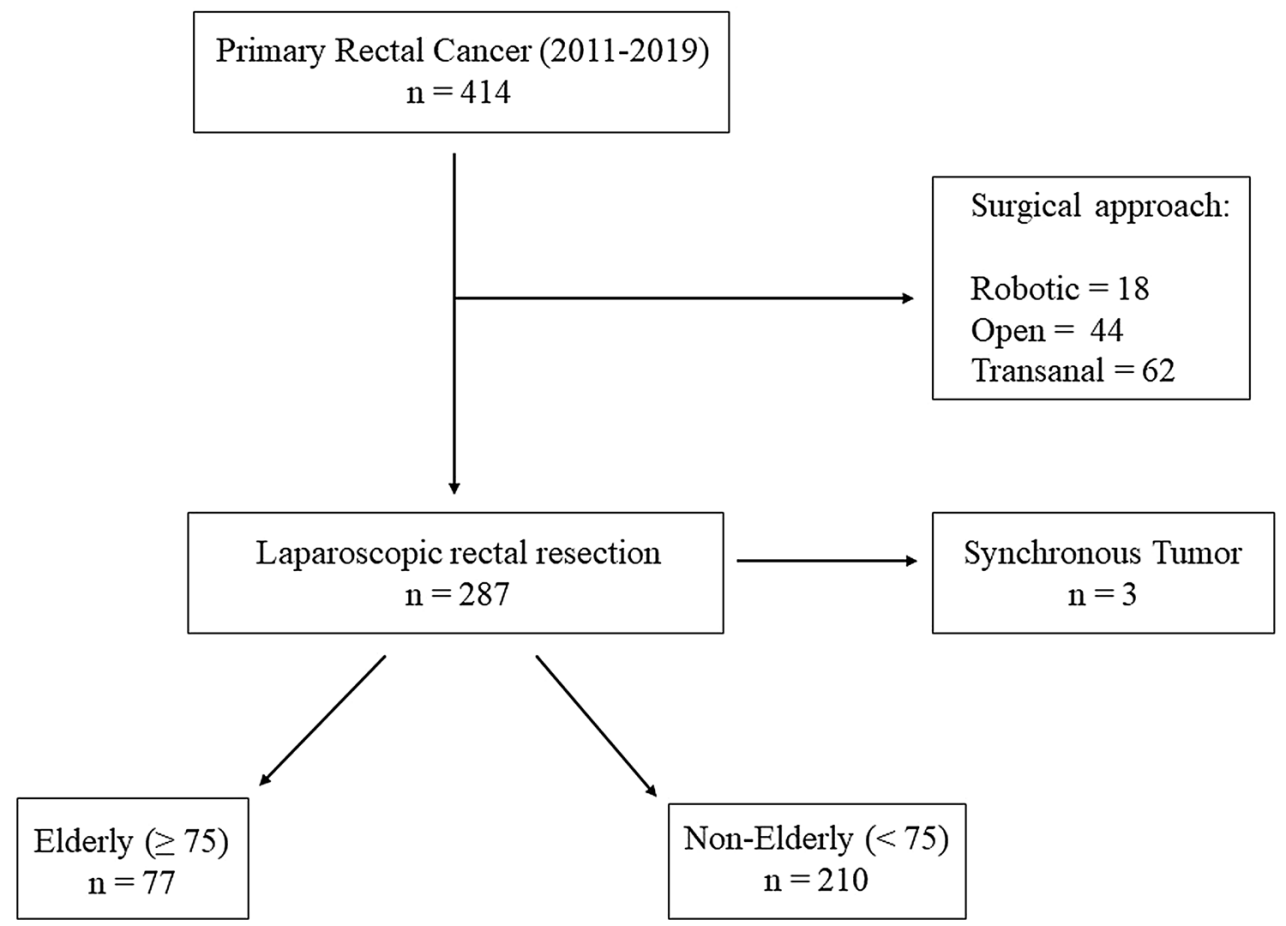
Flowchart of patients selection

Consecutive unselected patients with primary rectal cancer submitted to elective laparoscopic anterior resection were enrolled in the study. Patients undergoing the same surgical procedure with open, robotic or transanal approach and those undergoing abdominal perineal resection or local excision by transanal endoscopy microsurgery (TEM) or transanal minimally invasive surgery (TAMIS) were excluded. Furthermore, other exclusion criteria was synchronous neoplasia (Fig. 1).

Preoperative data regarding demographic and disease characteristics were extracted from the databases. Age, gender, BMI, associated comorbidities and previous surgeries or neoadjuvant treatment, were recorded as well as the American Society of Anaesthesiologists (ASA) score divided into two categories (ASA I-II and ASA III-IV). Tumor location was classified as high, medium and low when the distance from its lower edge to anal verge was between 10.1–15 cm, 5.1–10 cm and 0–5 cm respectively [40] while staging followed the American Joint Committee on Cancer (AJCC)/TNM system (8^th^ edition) [41].

Postoperative complications have been reported during the postoperative hospital stay and within 30 days of surgery, including the anastomotic leakage (AL) rate with related treatments and the length of hospitalization. AL was defined as a defect of the intestinal wall at the anastomotic site evaluated by CT scan or endoscopy. Finally, a univariate and multivariate analysis of demographic, clinical and perioperative factors was performed to identify the independent variables related to postoperative complications. In particular, the analysis was conducted with patients’ stratification into groups based on age and the presence of associated comorbidities.

### Surgical procedure

All patients underwent laparoscopic surgery under general anaesthesia and preoperative chemoradiotherapy in case of locally advanced tumors (T3—T4 and/or N +) of the middle/lower rectum. Anterior resection with partial mesorectal excision (PME) was performed for tumors of the upper rectum. When the neoplasm involved the middle and lower third of the rectum, a total mesorectal excision (TME) was performed according to international guidelines [1, 42] with a temporary protective loop ileostomy. A mechanical anastomosis was performed by double stapling technique or alternatively a manual coloanal anastomosis and the specimen was extracted through a suprapubic incision. Conversion was defined as the need to perform a conventional laparotomy to perform the procedure or a premature abdominal incision for dissection or vascular control. All procedures were performed by surgeons experienced in colorectal surgery.

### Statistical analysis

The descriptive statistics used included determination of mean values and standard deviation (SD) or median values and interquartile range (IQR) of the continuous variables, and of percentages and proportions of the categorical variables.

Statistical analysis was performed using Chi-square, Fisher’s exact test, Student’s *t* test test and ANOVA, where appropriate.

Binary logistic regression was used to examine the relationship between the presence of postoperative complications as a dependent variable and the possible predictors as independent variables. The following variables were included in the univariable analysis: male gender (vs. female), age at surgery (< 75 vs.  ≥ 75 and < 64 vs. 65–74 vs. 75–84 vs.  > 85), ASA status (ASA1-2 vs. 3–4), comorbidities (diabetes yes/no, COP yes/no, hypertension or cardiovascular diseases yes/no), previous surgery (yes vs not), smoking habits (yes/no), BMI (< 24,9 vs. 25–29,9 vs.  > 30), tumor location (mid-low vs high), T stage (T1 vs. T2 vs. T3 vs. T4), neoadjuvant chemoradiation (yes/no), type of surgery (PME vs. TME), conversion to open surgery (yes/no). The multivariable analysis was performed using the stepwise backward method (Wald) and it included all the variables with a *p* < 0.1 at univariable analysis. The coefficients obtained from the logistic regression analysis were also expressed in terms of odds of event occurrence (odd ratio—OR). A *p* value of less than 0.05 was considered statistically significant.

Data were analysed using the Statistical Package for Social Sciences (SPSS software v.15.0, Chicago IL, United States) for Windows and StatsDirect statistical software (vers. 3.0 StatsDirect, London, UK).

## Results

### Demographics and intraoperative data

A total of 287 patients underwent laparoscopic anterior rectal resection in three different institutions between April 2011 and July 2020. Patients aged < 75 were 210 and patients aged ≥ 75 were 77. In the first group, the mean age was 62.04 ± 8.75 years while in the second group was 80.11 ± 3.29 years (*p* < 0.001) while 58.6% of patients under 75 and 62.3% among over 75 were male and, in both groups, the mean BMI was 25. The preoperative characteristics of the patients are reported in Table 1. No statistically significant differences between groups were noted for the location of the cancer, the preoperative T stage and the proportion of patients who underwent neoadjuvant chemoradiation. The mean age and frequency of patients with hypertension and cardiovascular disease was significantly higher in the over 75 group (*p* = 0.02). Additionally, ASA score was significantly higher in the elderly than in the group of relatively younger patients (*p* < 0.001).

**Table 1:** Demographics and preoperative data

	Age < 75 *n* = 210	Age ≥ 75 *n* = 77	*p*
Age	62.04 ± 8.75	80.11 ± 3.29	< 0.001
Male gender	123 (58.6%)	48 (62.3%)	0.6
BMI (kg/m^2^)	25.35 ± 9.21	25.74 ± 8.89	0.7
Comorbidity			
HT and/or CVD	93 (44.3%)	46 (59.7%)	0.02
COPD	15 (7.1%)	11 (14.3%)	0.1
Diabetes	22 (10.5%)	11 (14.3%)	0.5
Current smoking	22 (10.5%)	16 (20.8%)	0.3
Previous abdominal surgery	56 (26.7%)	20 (25.9%)	0.9
ASA			
1–2	135 (64.3%)	21 (27.3%)	< 0.001
3–4	75 (35.7%)	56 (72.7%)	< 0.001
Rectal cancer location			
High	75 (35.7%)	32 (41.5%)	0.4
Mid	95 (45.2%)	28 (36.4%)	0.2
Low	40 (19.1%)	17 (22.1%)	0.7
Preoperative T stage			
T1	25 (11.9%)	8 (10.4%)	0.9
T2	40 (19.1%)	13 (16.9%)	0.8
T3	129 (61.4%)	53 (68.8%)	0.3
T4	16 (7.6%)	3 (3.9%)	0.4
Preoperative CHR			
Yes	100 (47.6%)	34 (44.2%)	0.7
No	110 (52.4%)	43 (55.8%)	0.7

*BMI* body mass index, *HT* hypertension, *CVD* cardiovascula disease, *COPD* chronic obstructive pulmonary disease, *ASA* American society of anesthesiologists, *CHR* chemoradiation

Intraoperative data are shown in Table 2. One hundred and forty (66.7%) and 63 (68.8%) anterior resections with TME and 70 (33.3%) and 24 (31.2%) anterior resections with PME were performed in the under 75 and over 75 groups, respectively. In both cases, no significant differences were found. A protective loop ileostomy was carried out in almost half of the cases in both groups. Furthermore, the difference between the two groups in the conversion rate to open surgery was not significant (5.7% vs 10.4%; *p* = 0.3).

**Table 2:** Operative data

	Age < 75 *n* = 210 (%)	Age ≥ 75 *N* = 77 (%)	*p*
Type of surgery			
Anterior resection + PME	70 (33.3%)	24 (31.2%)	0.8
Anterior resection + TME	140 (66.7%)	53 (68.8%)	0.8
Protective ileostomy	96 (45.7%)	42 (54.5%)	0.2
Anastomosis type			
Stapled	194 (92.4%)	72 (93.5%)	0.9
Hand-sewn	16 (7.6%)	5 (6.5%)	0.9
Conversion to open surgery			
Yes	12 (5.7%)	8 (10.4%)	0.3
No	198 (94.3%)	69 (89.6%)	0.3

*PME* partial mesorectal excision, *TME* total mesorectal excision

### Postoperative outcomes

Details of postoperative recovery outcomes are summarized in Table 3.

**Table 3:** Postoperative outcomes

	Age < 75 *n* = 210 (%)	Age ≥ 75 *n* = 77 (%)	*p*
Complications			
Wound infection	14 (6.7)	7 (9.1)	0.6
Nausea and vomiting	2 (0.9)	2 (2.6)	0.6
Ileus/bowel obstruction	12 (5.7)	3 (3.9)	0.7
Bleeding	6 (2.9)	5 (6.5)	0.3
Pulmonary	4 (1.9)	5 (6.5)	0.1
Cardiovascular	12 (5.7)	5 (6.5)	0.9
Urologic	3 (1.4)	1 (1.3)	0.6
Renal	3 (1.4)	3 (3.9)	0.4
Neurologic	2 (0.9)	2 (2.6)	0.6
Electrolyte imbalance	2 (0.9)	1 (1.3)	0.7
Sepsis	1 (0.5)	0 (0)	0.6
Ileum perforation	0 (0)	1 (1.3)	0.6
Anastomotic leakage	32 (15.2)	5 (6.5)	0.07
Overall complications	93 (44.3)	40 (51.9)	0.3
Patients with complications	68 (32.4)	31 (40.2)	0.2
Anastomotic leakage treatment			
Antibiotics and/or drainage	16/32 (50)	3/5 (60)	0.7
Stoma	7/32 (21.9)	0 (0)	0.2
Redo anastomosis	9/32 (28.1)	2/5 (40)	0.2
Postoperative blood transfusion	8 (3.8)	6 (7.8)	0.3
LOS (days), median (IQR)	7 (4)	7 (2.75)	0.2
Death during hospitalization	2 (0.9)	0 (0)	0.9

*LOS* length of hospital stay, *IQR* interquartile range

The overall postoperative complication rate was not significantly different between the two groups (44.3% vs. 51.9%; *p* = 0.3), as well as the rate of patients who developed at least one complication (32.4% vs. 40.2%; *p* = 0.2). The incidence of anastomotic leakage was, respectively, 15.2% and 6.5% in the under 75 and over 75 group (*p* = 0.07) and no differences were recorded in the management of this specific complication, as well as in the need for postoperative red blood cells (RBC) transfusions. During hospitalization, only two patients died both in the under 75 group (0.9%). The mean hospital stay was 7.0 (4.0) and 7.0 (2.75) days in the two groups (*p* = 0.2).

As shown in Table 4, the age of patients stratified into classes is not related to the risk of postoperative complications as well as previous comorbidities, BMI, neoadjuvant treatment, type of intervention and conversion to open during the procedure. The only independent predictive variables are represented by the male gender (OR 2.56; 95% CI 1.53–3.68, *p* < 0.01) and by the low-medium localization of the tumor (OR 2.12; 75% CI 1.43–4.21, *p* < 0.01).

**Table 4:** Univariate and multivariate analysis of variables associated with postoperative complications

	Univariate analysis			Multivariate analysis	
	No complications (*n* = 188)	Complications (*n* = 99)	*p* value	OR (95% CI)	*p* value
Gender					
Male	101 (53.7%)	70 (70.7%)	0.007	2.56 (1.53–3.68)	< 0.01
Age					
< 75	142 (75.5%)	68 (68.7%)	0.3		
≥ 75	46 (24.5%)	31 (31.3%)	0.3		
Age subgroups					
< 64	80 (42.6%)	30 (30.3%)	0.04	0.89 (0.56–2.12)	0.09
65–74	62 (33%)	38 (38.4%)	0.36		
75–84	42 (22.3%)	26 (26.3%)	0.4		
≥ 85	4 (2.1%)	5 (5%)	0.17		
ASA score					
I-II	107 (56.9%)	49 (49.5%)	0.22		
III-IV	81 (43.1%)	50 (50.5%)	0.23		
Comorbidities					
HT and/or CVD	84 (44.7%)	55 (55.6%)	0.1		
Diabetes	21 (11.2%)	12 (12.1%)	0.6		
COPD	15 (8%)	11 (11.1%)	0.5		
At least 1 comorb	99 (52.7%)	61 (61.6%)	0.2		
At least 2 comorb	17 (9%)	16 (16.2%)	0.1		
At least 3 comorb	3 (1.6%)	1 (1%)	0.9		
Previous surgery	52 (27.7%)	24 (24.2%)	0.7		
Smokers	62 (33%)	38 (38.4%)	0.4		
BMI (kg/m^2^)					
< 24.9	89 (47.3%)	43 (43.4%)	0.2		
25–29.9	73 (38.8%)	41 (41.4%)	0.7		
> 30	26 (13.8%)	15 (15.2%)	0.9		
Tumor location					
Mid-Low	109 (58%)	71 (71.7%)	0.03	2.12 (1.43–4.21)	< 0.01
T stage					
T1	26 (13.8%)	7 (7.1%)	0.08	0.84 (0.49–2.67)	0.2
T2	37 (19.7%)	16 (16.2%)	0.4		
T3	116 (61.7%)	66 (66.6%)	0.4		
T4	9 (4.8%)	10 (10.1%)	0.08	1.73 (0.86–3.22)	0.3
Neoadiuvant CHR					
Yes	81 (43.1%)	53 (53.5%)	0.11		
No	107 (56.9%)	46 (46.5%)	0.11		
Type of surgery					
Anterior resection + PME	66 (35.1%)	28 (28.3%)	0.3		
Anterior resection + TME	122 (64.9%)	71 (71.7%)	0.3		
Conversion to open surgery	14 (7.4%)	6 (6.1%)	0.8		

## Discussion

The rate of surgical resections for rectal cancer has significantly decreased over the years in patients ≥ 75 years of age [43]. This is partly due to higher comorbidity prevalence of patients [4], but also to the development of conservative treatment options showing remarkable results [44, 45], although there are still some controversial aspects and a limited application of these alternative treatments to current clinical practice [46, 47]. Surgery still remains the main 'cornerstone' for the treatment of rectal cancer, demonstrating a progressive implementation of minimally invasive techniques with acceptable oncological and functional outcomes [48]. In this setting, laparoscopy has proven to be safe, advantageous and an effective alternative to open surgery even in elderly patients with colorectal cancer [49], as well as for benign diseases [50]. Thus, the next step was to assess whether there was a difference in short-term outcomes after laparoscopic surgery for colorectal cancer between the elderly and non-elderly population. A recent meta-analysis finds a higher overall complication rate in elderly patients aged ≥ 75 years undergoing laparoscopic colorectal resections (*p* < 0.01) [18] likewise of the open approach [3]. However, the review includes both colonic and rectal laparoscopic resections. Since few studies exclusively considered rectal surgery or separately reported data after laparoscopic rectal cancer surgery in the elderly patients, [19, 20, 51, 52], the aim of the present study was to assess whether a more vulnerable population, consisting of older people, can benefit from a minimally invasive surgical approach for the treatment of rectal cancer in the same way as relatively younger people by evaluating age groups and individual or overall comorbidities as possible indipendent risk factor.

In this study, although older patients have both the ASA score and the prevalence of cardiovascular disease significantly higher than the non-elderly patients, the postoperative complication rates and the number of patients with complications between the over-75 group and the under-75 are comparable. Also, there were no significant differences in length of hospital stay and mortality rate.

The leakage rate after anterior rectal resection ranges from 3 to 23% [53]. In the present study, the incidence of anastomotic leakage is higher in the cohort of patients aged < 75 years, although it does not reach a statistically significant difference (15.2% vs. 6.5%; *p* = 0.07). This discrepancy can be attributed to various intraoperative risk factors such as long operative time, the number of stapler firings and anastomotic level that are associated with increased risk of leakage [54, 55], but which were not taken into account in the analysis. Treatment was mainly carried out by relaparoscopy given the great experience of the centers [56].

Finally, the results of multivariate regression analysis show that only male gender and low-mid rectal tumor localization are independent risk factors related to postoperative morbidity, whereas age and associated comorbidities did not have an impact on complications. These findings suggest that the laparoscopic approach for rectal cancer surgery is safe and appropriate even for patients aged ≥ 75 years, by demonstrating a rate of adverse events after surgery similar to that of patients under 75.

The results of the present study is consistent with the few previous reports that compared the safety and feasibility of laparoscopic rectal resection of the elderly with younger patients and they may be useful in clinical practice if interpreted wisely to mitigate the risk of conversion [57]. From Table 5, only one study exclusively focuses on rectal cancer surgery [19], two of them reported and compared preoperative patients’ data [19, 20] and no logistic regression analysis was performed in any study to identify predictors of complications. Therefore, only in the present case series, the age of patients divided into groups and the impact of individual and overall preoperative comorbidity were systematically excluded as possible independent risk factors of postoperative complications, assuming that laparoscopic surgery should be a valid choice for the elderly patient with rectal cancer because of an overall complication rate comparable to rate non-elderly patients, unlike open surgery [3] or as reported elsewhere [18].

**Table 5:** Studies comparing laparoscopic rectal surgery in the elderly patients vs. non-elderly patients

Authors	Year	Setting	Groups	*n* patients	Significative difference for ASA score	Overall complications (%)	*p*	LOS (days)	*p*	Mortality (%)	*p*
Scheidbach et al. [52]	2005	Multicentric	> 75	193	NR	54 (55.7)	NS	NR		5 (5.2)	NS
			< 75	508		179 (71.6)				0	
Chautard et al. [51]	2008	Single center	≥ 70	27^a^	NR	9 (33)	NS	15 (6–63)	NS	0	
			< 70	34^b^		15 (44)		15 (6–75)		0	
Akiyoshi et al. [19]	2009	Single center	≥ 75	44	Yes	6 (13.6)	NS	19 (7–123)	NS	0	
			< 75	228		27 (11.8)		15 (5–55)		0	
Roscio et al. [20]	2016	Two centers	> 80	33	Yes	21 (63.6)	NS	8 (8–9)	NS	0	
			60–69	82		43 (52.4)		8 (7–9)		0	

*NR* not reported, *NS* not significative

^a^22 rectal cancer

^b^24 rectal cancer

It has been demnostrated that laparoscopy and robotic surgery have similar effectiveness in oncologic outcomes, but robotic surgery may have lower conversion rates compared to laparoscopy especially in patients with high BMI, lower lesions and after neodjuvant [58]. However, it also true that laparoscopic rectal cancer resection in selected and fit patients and in high-volume centers with laparoscopic expertise can achieve safe oncological outcomes and margins with sphincter-sparing dissection, even in ultralow rectal cancers and without needing of robotic surgery or transanal TME (TaTME) [59]. In this setting, robotic TaTME seems a promising apporach to improve the outcomes and feasibility of low rectal cancers resections but this technique, although recently described [60] is still considered too preliminary by some authors and can not be recommended as yet [61].

This study has several limitations. The absence of a satisfactory matching process limits the risk of bias regarding its retrospective design as well as the limited number of patients over 75, although data comes from multiple centers. In addition, the long-term oncological outcomes and data relating to the Enhanced Recovery After Surgery protocol (ERAS) are not included. Furthermore, the choice of a laparoscopic approach rather than others to performe rectal resection was at the discretion of each surgical team increasing the risk of selection bias. However, the analysis of data collected from high-volume laparoscopic colorectal surgery centers suggests that patients over 75 years old benefit of a laparoscopic approach as well as younger patients despite advanced age and previous comorbidities.

These findings may depend on the fact that the most problematic expression of population aging would not be the age ot the comorbidities, but the clinical condition of frailty, which is defined as “a state of increased vulnerability to poor resolution of homoeostasis after a stressor event, which increases the risk of adverse outcomes, as a consequence of cumulative decline in many physiological systems during a lifetime” [62]. However, it is believed that this cannot significantly compromise the results of the present study. In fact, a systematic review of the literature shows that the prevalence of frailty increases with age [63] and according to some authors the concept of fragility is closely related to comorbidity and frequently overlaps with it [64]. In addition, there is no clear consensus on the definition to date. On the one hand, it is considered only a phenotype of fragility exclusively linked to the physical condition; on the other, it is considered more appropriate to extend its definition to include social and psychological aspects [63]. Finally, several screening methods have been developed to predict the degree of frailty in elderly patients with cancer, but none have demonstrated sufficient discriminatory power, stating that a comprehensive geriatric assessment is the most valid modality. [65]. However, a multidisciplinary holistic assessment of the elderly patient in the perioperative period remains desirable.

## Conclusions

Despite higher incidence of cardiovascular disease and a higher anesthesiologic risk, short-term postoperative outcomes in patients ≥ 75 years of age are similar to those of younger patients after laparoscopic surgery for rectal cancer. Advanced age and preoperative evaluated comorbidities are not related to an increased risk of postoperative morbidity, unlike open surgery. Therefore they should not represent a limitation to laparoscopic rectal resection.
